# Stars and stripes in pancreatic cancer: role of stellate cells and stroma in cancer progression

**DOI:** 10.3389/fphys.2014.00052

**Published:** 2014-02-14

**Authors:** Jeremy S. Wilson, Romano C. Pirola, Minoti V. Apte

**Affiliations:** ^1^Pancreatic Research Group, Faculty of Medicine, South Western Sydney Clinical School, Ingham Institute for Applied Medical Research, University of New South WalesLiverpool, NSW, Australia; ^2^Ingham Institute for Applied Medical ResearchLiverpool, NSW, Australia

**Keywords:** pancreatic cancer, pancreatic stellate cells, desmoplastic/stromal reaction, stromal-tumor interactions, stromal therapeutic targets

## Abstract

Pancreatic cancer is a devastating disease with an unacceptably high mortality to incidence ratio. Traditional therapeutic approaches such as surgery in combination with chemo- or radiotherapy have had limited efficacy in improving the outcome of this disease. Up until just under a decade ago, the prominent desmoplastic reaction which is a characteristic of the majority of pancreatic ductal adenocarcinomas (PDAC) had been largely ignored. However, since the identification of the pancreatic stellate cell (PSC) as the key cell responsible for the production of the collagenous stroma in PDAC, increasing attention has been paid to the role of the stromal reaction in pancreatic cancer pathobiology. There is now compelling evidence that PSCs interact not only with cancer cells themselves, but with several other cell types in the stroma (endothelial cells, immune cells, and possibly neuronal cells) to promote cancer progression. This review summarizes current knowledge in the field about the influence of PSCs and the stromal microenvironment on cancer behavior and discusses novel therapeutic approaches which reflect an increasing awareness amongst clinicians and researchers that targeting cancer cells alone is no longer sufficient to improve patient outcome and that combinatorial treatments targeting the stroma as well as the cancer cells will be required to change the clinical course of this disease.

## Introduction

Pancreatic cancer (pancreatic ductal adenocarcinoma; PDAC) is a lethal disease. It is the fourth leading cause of cancer related death in developed countries (Jemal et al., 2011; Siegel et al., 2013). Five year survival is at best 6% and survival beyond 12 months is unusual. Only 20% of patients are deemed suitable for attempted curative resection. Chemotherapy confers marginal benefit while the benefit of radiotherapy is debated. There are several reasons for this grim outlook. As the pancreas is a retroperitoneal organ, cancers in its body and tail present late, often with considerable local and distant spread. Early symptoms are often non-specific. There are no biomarkers for the disease.

Risk factors for pancreatic cancer include age, smoking, race, diabetes, and chronic pancreatitis. The strongest known risk factor for pancreatic cancer is chronic pancreatitis. Patients with a history of more than 5 years chronic pancreatitis have a greater than 14-fold risk of developing pancreatic cancer compared to the general population (Chu et al., 2007; Pandol et al., 2012). A significant proportion (40%) of patients with hereditary pancreatitis is at increased risk of developing pancreatic cancer (Whitcomb and Greer, 2009). For patients with tropical pancreatitis, a 100-fold increased risk and an earlier onset of pancreatic cancer has been reported (Chari et al., 1994; Whitcomb, 2004). The mechanisms underlying this increased propensity for patients with chronic pancreatitis to develop pancreatic cancer are not fully elucidated although recent studies suggest that several signaling pathways known to be active in inflammatory disease may be involved in driving this process (Thomasova et al., 2012).

**Figure 1:**
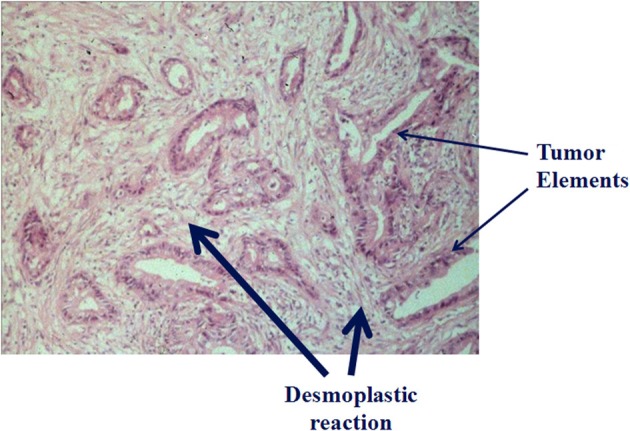
**Histology of pancreatic cancer: *Hematoxylin* and eosin stained section of a human pancreatic cancer section showing tumor elements (thin arrows) embedded in an abundant collagenous stroma (thick arrows) (*previously unpublished figure*)**.

Histologically, PDAC is characterized by an extensive and dense desmoplastic/fibrotic stroma in which cancer cells are embedded (Figure 1). It has now been unequivocally shown that the principal effector cells responsible for the production of this stroma are pancreatic stellate cells (PSCs) (Apte et al., 2004). Considerable evidence has also accumulated in recent years to indicate that this abundant stroma can no longer be considered a mere bystander in pancreatic cancer pathobiology, but should be recognized as a critical player in cancer progression.

This review will concentrate on the interactions between PSCs and pancreatic cancer cells and will also touch upon recent reports about the interactions between PSCs and other stromal cells (endothelial, immune, and nerve cells), all of which have the potential to influence local growth and distant spread of pancreatic tumors.

## Pancreatic stellate cells (PSCs)

PSCs were first described by Watari et al. (1982). These resident cells of the pancreas are predominantly periacinar in location and comprise 4–7% of total pancreatic parenchymal cells. In the healthy pancreas, PSCs are in a quiescent state and exhibit abundant vitamin A containing lipid droplets in their cytoplasm (Apte et al., 1998). Similar cells exist in the liver—hepatic stellate cells (HSCs). HSCs were first described by Kupffer in 1876 but were brought into modern prominence by the work of Ito (1951) and Wake et al. (1987). Since that time, HSCs have been acknowledged as the principal site of storage of vitamin A in the body as well as being (when activated) the principal effector cells of liver fibrosis. It is now well-established that HSCs have a range of functions encompassing extracellular matrix (ECM) homeostasis, fibrosis, retinoid metabolism, liver development and regeneration, and immunomodulation (Lee and Friedman, 2011).

PSCs were first isolated by Apte et al. (1998) and this achievement opened up the field of pancreatic fibrogenesis as the cells could now be studied *in vitro* and *in vivo*. Since 1998, PSCs have been extensively characterized and their roles in fibrogenesis and tumor stromal interactions have been delineated in some detail (Apte et al., 2012).

**Figure 2:**
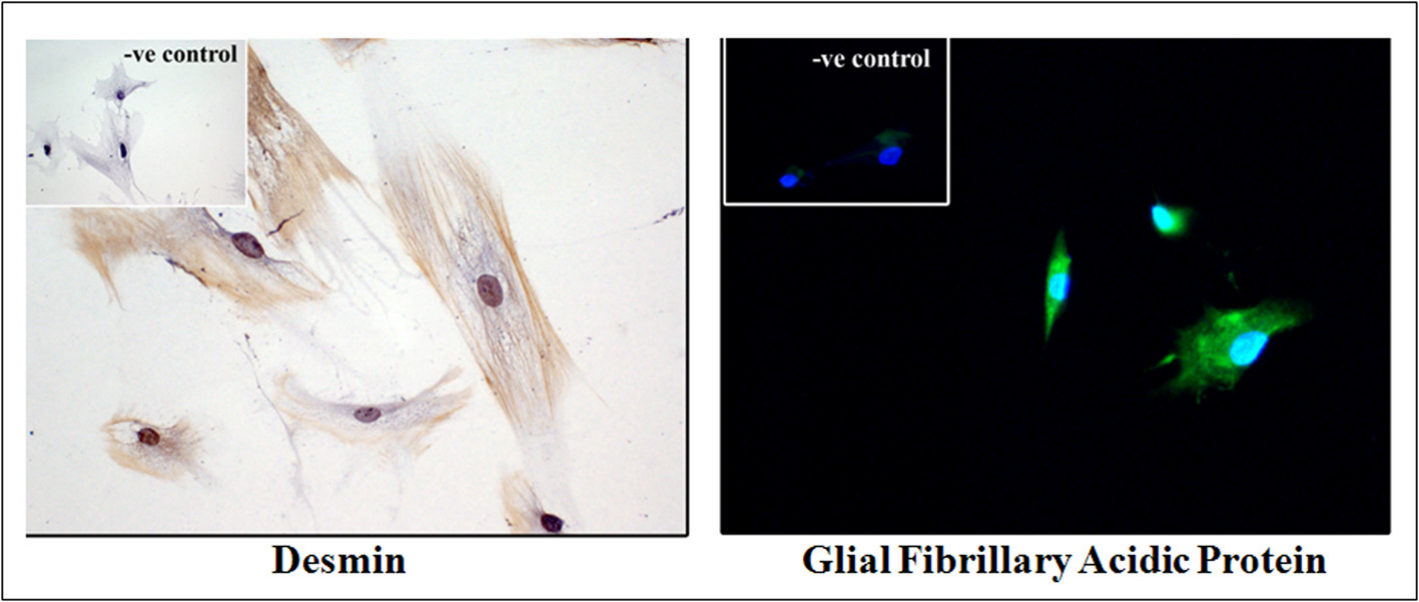
**Human pancreatic stellate cells (PSCs) in culture**. Immunocytochemical analysis of primary cultures of human PSCs exhibiting desmin and glial fibrillary acidic protein (GFAP) staining. Insets: Negative control (*previously unpublished figure*).

PSCs express desmin, glial fibrillary acid protein (GFAP), vimentin, and nestin (intermediate filament proteins) as well as the neuroectodermal markers such as nerve growth factor (NGF) and neural cell adhesion molecule; the expression of these selective markers differentiates PSCs from fibroblasts (Figure 2). At the ultrastructural level they feature a prominent rough endoplasmic reticulum, collagen fibrils, and lipid droplets surrounding a central nucleus. With their ability to produce ECM proteins as well as the enzymes that degrade ECM proteins [matrix metalloproteinases (MMPS)], and inhibitors of MMPs [tissue inhibitors of metalloproteinases (TIMPS)], PSCs are thought to play a primary role in maintenance of normal pancreatic architecture. However, when activated, during pancreatic injury, the cells lose their lipid droplets, express α-smooth muscle *actin* (α-SMA), proliferate, migrate, and produce excessive amounts of ECM proteins, resulting in a loss of the balance between ECM production and degradation and leading eventually to fibrosis. During an acute episode of pancreatic injury, PSCs are activated early, and secrete excess ECM proteins that lay down a lattice for regenerating epithelial cells. As the injury resolves, activated PSCs are lost most likely through apoptosis (Tahara et al., 2008; Vonlaufen et al., 2011). MMPs secreted by the remaining PSCs degrade the excess fibrosis resulting in restitution of normal pancreatic histology. However, with repeated or sustained injury, PSCs can attain a perpetually activated state, since the cells can secrete their own cytokines and growth factors, which in turn can activate PSCs via selected receptors on the cell surface (Mews et al., 2002; Masamune et al., 2009). Thus, even in the absence of the original triggers, PSCs can remain in their activated state eventually being responsible for the development of pathological, often irreversible fibrosis.

While most of the initial research attention was directed toward elucidating the mechanisms responsible for PSC-mediated pancreatic fibrosis, it is becoming increasingly clear that PSCs may have several additional functions in health and disease. These include:

- Role in pancreatic exocrine secretion: The secretagogue cholecystokinin (CCK) has been shown to directly stimulate exocrine secretion from rodent pancreatic acinar cells by binding to CCK receptors on the cell surface. However, there has been some controversy in the published literature regarding the direct effects of CCK on human pancreatic acinar cells, with Ji et al. reporting in 2001 and 2002 (Ji et al., 2001, 2002) that human acinar cells did not exhibit functional CCK receptors, a finding that was later countered by Murphy et al. (2008) who reported that isolated human pancreatic acini responded to physiological CCK concentrations by exhibiting the expected oscillatory rise in cytosolic calcium and by secreting amylase. In view of the close association of PSCs with the basolateral aspects of acinar cells, it has been postulated that in the human pancreas, PSCs may provide an alternative/additional pathway for CCK-mediated enzyme secretion by acting as intermediary cells in the CCK-stimulated secretory pathway. This concept is supported by the observations that (a) PSCs express both types of (CCK) receptors; (b) upon exposure to CCK, PSCs synthesize and secrete acetylcholine which can then act on muscarinic receptors on acinar cells leading to digestive enzyme release; and (c) PSC-mediated amylase secretion by acinar cells can be inhibited by the muscarinic receptor antagonist, atropine (Phillips et al., 2010).
- Role in innate immunity (Masamune et al., 2008a; Shimizu et al., 2012): PSCs express Toll-like receptors (TLR 2, 3, 4, 5, and 9) which recognize foreign pathogen-associated molecular patterns (PAMPs) and have been shown to be able to phagocytose necrotic and apoptotic cells. These functions suggest that the cells may have an “innate” immune function which protects local parenchyma, thereby limiting tissue damage during early pancreatic injury. However, the role of PSCs in acquired immunity is not as clear. Unlike their hepatic counterparts, PSCs do not express any antigen-presenting cell markers such as MHC class II or HLA-DR molecules. The reason for this difference between HSCs and PSCs is not known, but may reflect the fact that HSCs are routinely exposed to numerous antigens via the portal circulation resulting in the cells acquiring functions of antigen presenting cells, while PSCs are relatively protected within the pancreas.
- Role as progenitor cells (Mato et al., 2009; Kordes et al., 2013): Mato et al. (2009) used mitoxantrone (a compound that acts through multidrug transport systems) to isolate and expand a population of mitoxantrone-resistant pancreatic cells from lactating rats. They reported that these selected cells exhibited a morphology identical to PSCs, with vitamin-A containing lipid droplets in the cytoplasm. The cells also expressed ABCG2 transporter (ATP-binding cassette G2 transporter—a stem cell marker) and when incubated with an appropriate differentiating medium, were able to secrete insulin. More intriguingly, a recent study by Kordes et al. (2013) has reported that clonally expanded rat PSCs, when injected into hepatectomized recipient rats, were able to migrate to the liver and to reconstitute large parts of the liver by differentiating into hepatocytes and cholangiocytes, whereas muscle fibroblast did not show any such transformations.
- Role in cancer progression (Apte et al., 2013): There is now incontrovertible evidence from both *in vivo* and *in vitro* studies for a central role for PSCs in promoting local growth of pancreatic tumors as well as facilitating regional and distant spread of pancreatic cancer cells.

## Interactions between PSC and pancreatic cancer cells, endothelial cells, immune cells, and neural cells

### Evidence from *in vivo* studies

The role of PSCs in pancreatic cancer biology was initially studied in xenograft models and more recently has been examined using transgenic animal models of the disease. The earliest study in this area was published by Bachem et al. (2005), who used a subcutaneous xenograft model in immunocompromised mice to demonstrate increased growth of pancreatic cancer cells when co-injected with PSCs into the flanks of mice. The tumors produced in mice injected with both cell types were significantly larger than those in mice injected with cancer cells alone, exhibiting increased fibrosis as well as enhanced cancer cell proliferation. These observations suggested that, in addition to producing the collagenous stroma, PSCs also directly stimulated cancer cell growth.

Although the above findings were of interest, it is well-known that subcutaneous xenograft models of pancreatic cancer have an important limitation—the natural tumor microenvironment is absent in these models. Therefore, orthotopic tumors produced by implantation/injection of cancer cells directly into the pancreas are a preferred option. Such cells would be exposed to the same microenvironment as may be expected in human pancreatic cancer and would also have the capacity to metastasize, further simulating the human condition.

**Figure 3:**
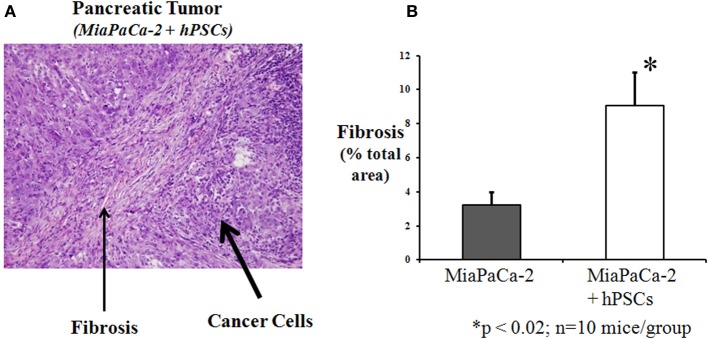
**Orthotopic pancreatic tumor produced by injecting a mixture of human pancreatic cancer cells (MiaPaCa-2) and human pancreatic stellate cells into the pancreas of nude mice. (A)** H and E staining of tumor section showing prominent areas of fibrosis (desmoplasia) within the tumor. Reprinted with permission Vonlaufen et al. (2008a). **(B)** Fibrosis was quantitated by morphometry of Masson's stained sections (not shown). The graph depicts the significant increase in fibrosis in tumors produced by injection of a mixture of PSCs and cancer cells (MiaPaCa-2), compared to cancer cells alone. ^*^p < 0.02; *n* = 10 mice/group (*previously unpublished data*).

In recent years, several studies have reported orthotopic models of pancreatic cancer involving direct implantation/injection into the mouse pancreas of human pancreatic cancer cells (MiaPaCa-2, BxPC-1, AsPC-1) with or without human PSCs (hPSCs) (Hwang et al., 2008; Vonlaufen et al., 2008a; Xu et al., 2010). The presence of hPSCs enhanced local tumor growth as well as regional and distant metastasis. Tumors composed of both cancer cells and hPSCs exhibited (i) bands of fibrosis (resembling desmoplasia) containing α-SMA positive (activated) PSCs (Figure 3); and (ii) increased proliferation and decreased apoptosis of cancer cells, suggesting that the presence of PSCs increased the survival of cancer cells. These observations concur with those seen with hPSCs and tumor cells *in vitro* (vide infra) and support a role for PSCs in pancreatic cancer progression.

Orthotopic tumors produced by cancer cells + PSCs also exhibited enhanced angiogenesis (as indicated by the upregulation of the endothelial cell marker CD31) compared to tumors produced by the injection of cancer cells only, suggesting that PSCs stimulate angiogenesis in pancreatic cancer (Xu et al., 2010). It must be noted here however, that angiogenesis in human pancreatic cancers may be more complex than that observed in orthotopic models. Indeed, the central areas of advanced pancreatic tumors in humans are known to be very poorly perfused and hypoxic, with only a few blood vessels evident on histological examination; it is only the invading front of the cancers that manifests neoangiogenesis (Erkan et al., 2009). These findings are supported by *in vitro* work indicating that while the inductive effect of PSCs on angiogenesis is well-demonstrated under normoxic conditions, the same cannot be demonstrated under hypoxic conditions (Erkan et al., 2009). Thus, the overall influence of PSCs on angiogenesis in pancreatic cancer (taking into account the differences in oxygenation within the tumor) remains to be fully clarified.

One of the well-documented features of human pancreatic cancer is its resistance to chemotherapeutic agents and to radiotherapy. It is possible that this resistance may be mediated, at least in part, by the dense stroma produced by PSCs (Hanahan and Weinberg, 2011). In support of this notion, it has been shown that sequestration of chemotherapeutic agents such as gemcitabine can occur in the tumor stroma, effectively reducing the amount of the drug that can reach cancer cells (Olive et al., 2009). Furthermore, Mantoni et al. (2011) have reported that PSCs protect cancer cells from radiation via a β 1-integrin dependent pathway.

As indicated above, in orthotopic models, PSCs have been shown to promote tumor metastasis. Traditionally, only cancer cells have been thought to possess metastatic capabilities, which allow the cells to intravasate into blood vessels or lymphatics, travel through the circulation, and extravasate at distant sites. This concept has been challenged by the findings of Xu et al. (2010) who, using a gender mismatch approach have demonstrated that PSCs from the primary tumor can also be detected at distant metastatic sites. The authors injected a mixture of male human PSCs and female cancer cells (AsPC-1 cell line from a female patient), into the pancreas of female mice. Using fluorescent *in situ* hybridization, y chromosome positive cells were detected not only in the primary tumors (as expected) but also within metastatic nodules in the mediastinum, liver, and diaphragm. These observations indicate that PSCs can travel to distant metastatic sites (possibly with cancer cells), where they may be reasonably postulated to play a role in the seeding, survival, and proliferation of cancer cells. A subsequent study reported similar findings in a model of lung cancer (Duda et al., 2010) suggesting that metastasis can no longer be considered the sole preserve of cancer cells.

In contrast to subcutaneous and orthotopic models where tumors are produced in immunocompromised mice by xenografts of human pancreatic cancer cells and PSCs, some genetically engineered mouse (GEM) models exhibit the development of spontaneous pancreatic cancer with a prominent endogenously produced stromal reaction (Guerra and Barbacid, 2013). These models include KPC mice (Kras^LSL−G12D/+^; Trp53^LSL−R172H/+^; Pdx^cre/+^), KPGC mice (Kras^LSL−G12D/+^; Trp53^LSL−R172H/+^; R26^LSL−GFP/+^; Pdx^cre/+^), and TGFβ type II receptor organ specific knockout in the mouse pancreas (Kras^LSL−G12D/+^; TGFβr2^floxflox^; Ptf1a^cre/+^). The lesions in these models progress from preinvasive ductal changes (PanIN lesions) to overt carcinoma and metastases, with an associated progressive increase in the surrounding stromal reaction. Importantly, activated PSCs have been observed in the earliest PanIN lesions (Ijichi et al., 2011; Apte et al., 2013). These GEM models provide an additional *in vivo* tool to assess the interactions between cancer cells and an endogenous stromal reaction and also to trial new therapeutic strategies in pancreatic cancer.

Evasion of the immune system is a well-recognized feature of pancreatic cancer (Bayne et al., 2012). Pancreatic cancer tissue is infiltrated with immune cells, such as T cells, B cells, NK cells, neutrophils, and macrophages as well as myeloid-derived suppressor cells (as the name suggests, MDSCs have a largely immunosuppressive function) (Apte et al., 2013; Ene-Obong et al., 2013; Hamada et al., 2013; Ino et al., 2013). Higher levels of CD8+ T cell infiltration have been shown to correlate with a better survival (Ene-Obong et al., 2013; Ino et al., 2013), while macrophage and neutrophil infiltration as well as high levels of MDSCs have been reported to be associated with poor survival (Gabitass et al., 2011; Ino et al., 2013). It has been demonstrated that cancer cells can evade the host immune system by producing granulocyte-macrophage colony-stimulating factor to suppress anti-tumor T cell immunity (Bayne et al., 2012).

Recent studies suggest that PSCs may also aid immune evasion. PSCs in the stroma of PanIN lesions and around cancer cells produce galectin-1, a β-galactoside-binding protein (Chen et al., 2012), that binds to N-acetyllactosamine on membrane glycoproteins and induces apoptosis in T cells thus suppressing the immune response (Tang et al., 2012). Ene-Obong et al. (2013) have reported that activated PSCs reduce the migration of CD8 positive T cells toward cancer cells in both human PDAC and the KPC mouse model of pancreatic cancer. Fibroblast activation protein-α (FAP-α), known to be expressed by stromal cells, is another protein that has been reported to disrupt anti-tumor immunity. Depletion of the cells expressing FAP-α enabled immune response-associated tumor regression, supporting the notion that FAP-α might act as an immune suppressor in pancreatic cancer (Kraman et al., 2010). Most recently, another type of immune cell, the mast cell, has been reported to play a role in pancreatic cancer progression. Using an orthotopic model of pancreatic cancer, Chang et al. (2013) have reported that cancer growth is significantly hampered in mast cell deficient Kit mice, while the reconstitution of mast cells in these mice from the bone marrow of wild type mice significantly enhanced tumor growth. Interestingly, as detailed later in this review, PSCs have been shown to activate mast cells *in vitro* (Ma et al., 2013), suggesting cross-talk between these two cell types in the stroma.

Taken together, the above studies suggest that PSCs may negatively modulate immune responses.

### Evidence from vitro studies

Findings derived from mouse models and observations on resected human tissue are supported by a number of *in vitro* studies which have confirmed a close bi-directional interaction between pancreatic cancer cells and PSCs.

When PSCs are exposed to cancer cells (either by co-culture or by using conditioned media), they are activated and manifest increased proliferation, migration, and ECM production (Apte and Wilson, 2012). In turn, PSCs stimulate cancer cell proliferation and inhibit cancer cell apoptosis thereby facilitating cancer cell survival (Vonlaufen et al., 2008b). PSCs have also been shown to promote cancer cell migration, during which cancer cells exhibit features of epithelial-mesenchymal transition (EMT) namely, decreased levels of epithelial markers such as E-cadherin concurrent with increased expression of mesenchymal markers (vimentin and Snail) (Fujiwara et al., 2013). It is possible that EMT is responsible (at least in part) for the PSC-induced increased migration of cancer cells. Most recently, a study by Bachem et al. (Lu et al., 2014) has demonstrated that PSC-induced cancer cell migration is dependent on collagen I secreted by PSCs; interaction of cancer cells with collagen I enhances the α2/β 1 integrin-focal adhesion kinase (FAK) signaling pathway that regulates migration of cancer cells.

While the above effects of PSCs on cancer cells are of significant interest, researchers have also been mindful of the known heterogeneity of pancreatic cancer with respect to rate of progression. This has led to studies examining whether all PSCs uniformly exert the same effects on cancer cells. Interestingly, a subpopulation of PSCs that express CD10 (a cell membrane associated matrix metalloproteinase), has been reported to induce significantly greater effects on cancer cell proliferation and invasion than CD10^−^ PSCs (Ikenaga et al., 2010). These findings indicate that functional heterogeneity between PSC populations may dictate the ultimate effects of these cells on cancer cell behavior.

One of the major factors responsible for the poor prognosis of pancreatic cancer is its propensity for recurrence, with recurrent tumors postulated to arise from a niche of drug resistant cancer stem cells. Recent evidence suggests that PSCs may play a role in facilitating such a stem cell niche in pancreatic cancer. Hamada et al. (2012) have reported that pancreatic cancer cells in co-culture with PSCs show increased expression of stem cell related genes such as nestin, ABCGZ, and LIN28, supporting the possibility that a PSC-facilitated cancer stem cell niche may be one of the factors responsible for recurrence of pancreatic cancer.

As the interactions between cancer cells and PSCs have become increasingly recognized, factors mediating these interactions have also attracted much interest. The increased proliferation of PSCs induced by cancer cells is likely mediated by platelet-derived growth factor (PDGF, a known mitogen for many cell types), which stimulates mitogen-activated protein kinase signaling (MAPK) in PSCs (Vonlaufen et al., 2008a). Recent studies have also implied that cancer cell-stimulated PSC proliferation is mediated by cyclooxygenase 2 (the inducible form of cyclooxygenases, which are enzymes involved in the conversion of arachidonic acid to prostaglandin Yoshida et al., 2005) and by trefoil factor 1 (a stable secretory protein that is upregulated in pancreatic cancer but is not expressed in normal pancreas) (Arumugam et al., 2011). The increase in ECM synthesis by PSCs upon exposure to cancer cells is thought to be mediated by transforming growth factor beta 1 (TGFβ 1) and fibroblast growth factor 2 (FGF2) (Bachem et al., 2005).

Factors mediating the effects of PSCs on cancer cells remain to be fully elucidated. Since cancer cells express receptors for PDGF and PSCs have the capacity to secrete PDGF, it has been postulated that this growth factor mediates the PSC-induced proliferation of cancer cells (Vonlaufen et al., 2008a). PSCs also secrete a cell adhesion protein named periostin, which has been found to increase the growth of cancer cells and their resistance to serum starvation and hypoxia (Erkan et al., 2007). Other candidate mediators that require further study include growth factors such as EGF, insulin-like growth factor (IGF), hepatocyte growth factor (HGF), and TGFβ as well as a variety of proinflammatory cytokines. Notably, ERK1/2 and Akt have been identified as the intracellular signaling pathways that regulate the response of cancer cells (increased migration, invasion, and colony formation) to PSC secretions (Hwang et al., 2008; Vonlaufen et al., 2008a).

The observed effects of PSCs on angiogenesis and metastatic spread *in vivo* (described earlier) are strongly supported by *in vitro* studies. PSCs have been shown to stimulate tube formation (a measure of angiogenesis) of human microvascular endothelial cells, an effect mediated by vascular endothelial growth factor (VEGF) secreted by PSCs (Xu et al., 2010). Under normoxic conditions, PSCs also induce endothelial cell proliferation, an effect again mediated by VEGF (Erkan et al., 2009). However, this proliferative effect of PSCs on endothelial cells was inhibited under hypoxic conditions (simulating the hypoxia in the center of a dense desmoplastic stroma), particularly in the presence of cancer cells (Erkan et al., 2009). On the other hand, hypoxia itself was shown to significantly increase PSC activation and ECM synthesis (Masamune et al., 2008b). Thus, the interplay between vessel density/oxygenation at different sites within the tumor (central vs. peripheral) and PSC activation, as well as the influence of PSCs on endothelial cell function under varying oxygen concentrations requires further study.

The ability of PSCs to travel from the primary tumor to metastatic sites (noted earlier) implies that PSCs can migrate through an endothelial layer. Using a Boyden chamber method with a porous membrane coated by a monolayer of endothelial cells, Xu et al. (2010) have shown that PSCs can invade and migrate through the endothelial cell layer, an effect that is enhanced in the presence of cancer cell secretions. This cancer cell-induced transendothelial migration of PSCs is mediated by PDGF in cancer cell secretions.

As noted earlier, pancreatic cancer cells have the ability to escape immune surveillance despite the presence of significant leukocyte infiltration in the stroma. There is *in vivo* evidence to suggest that PSCs may play a role in this immune evasion by sequestering CD8+ T cells and reducing their infiltration around tumor cells, thus preventing the T cells from exerting their anti-tumor effects. *In vitro* support for this concept comes from studies showing that PSCs exert a chemotactic effect on CD8+ T cells, and that this effect is mediated by the PSC-derived chemokine CXCL12 (Ene-Obong et al., 2013). Interactions between PSCs and mast cells have also been recently characterized (Ma et al., 2013). PSCs have been shown to activate mast cells *in vitro* promoting tryptase and IL13 release from the latter; these mast cell-derived factors have been shown to stimulate cancer cell proliferation. Mast cells also induce PSC proliferation, an effect mediated by IL13. Most recently, IL6 secreted by PSCs has been implicated in PSC-induced migration of the immunosuppressive cells MDSCs (Mace et al., 2013); as noted previously, high levels of MDSCs in pancreatic cancer tissue have been associated with reduced overall survival (Gabitass et al., 2011).

Compared to the interactions of PSCs with cancer cells, endothelial cells, and immune cells described above, little is known about the interaction of PSCs with neural elements in the desmoplastic reaction. However, extensive neural remodeling is known to occur in pancreatic cancer with the cancer stroma revealing neural hypertrophy and increased neural density (Ceyhan et al., 2010). It noteworthy that PSCs themselves express the neural markers GFAP and nestin, and also produce the neurotrophic factors NGF, brain-derived neurotrophic factor, and neurotrophin 45 (Haas et al., 2009; Demir et al., 2012). Thus, it would be reasonable to postulate that PSCs may act as neural elements in the tumor stroma, affecting the growth of nerves (via secretion of ECM components collagen and fibronectin and the neurotrophic factors noted above) and survival of cancer cells that express receptors for neurotrophic factors. This hypothesis is supported by a report by Ceyhan et al. (2009) demonstrating a positive correlation between the extent of desmoplasia and the degree of neural invasion in human PDAC.

**Figure 4:**
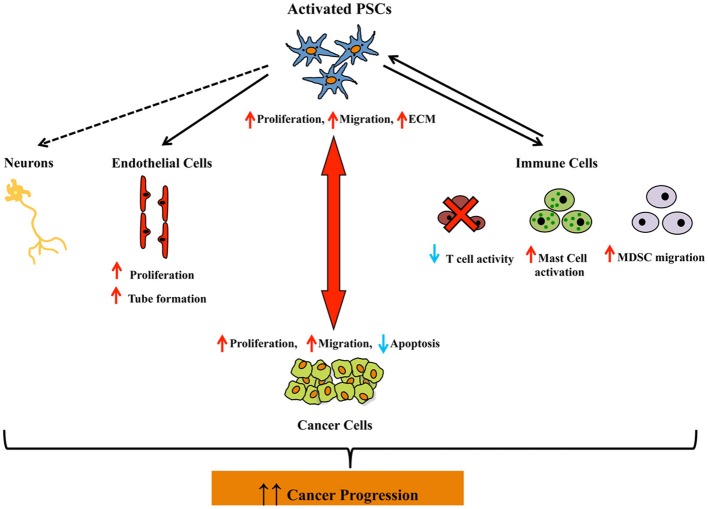
**Schematic diagram depicting the interactions between activated pancreatic stellate cells and pancreatic cancer cells, as well as pancreatic stellate cells and other stromal cells (endothelial cells, immune cells, and neuronal cells), all of which may promote pancreatic cancer progression**. The dashed arrow between PSCs and neuronal cells indicates that although an interaction between these two cell types may be reasonably postulated, direct experimental evidence in support of this concept has not yet been reported. Abbreviations: PSCs, pancreatic stellate cells; MC, mast cells; MDSC, myeloid derived suppressor cells; ECM, extracellular matrix.

Figure 4 summarizes the interactions between PSCs and pancreatic cancer cells as well as those between PSCs and other stromal cells that may promote cancer growth and spread.

## Do pancreatic stellate cells play a role in the earliest stages of pancreatic cancer?

**Figure 5:**
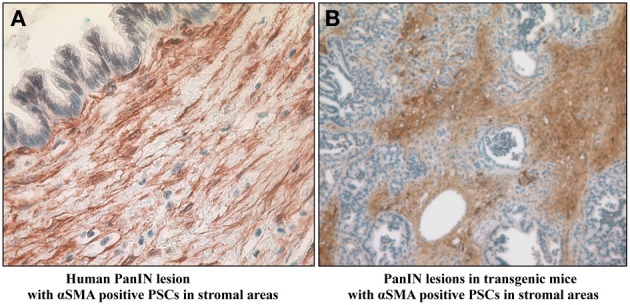
**Presence of α-smooth muscle actin positive activated pancreatic stellate cells in stroma surrounding early PanIN lesions in (A) humans and (B) transgenic mice**. Reprinted with permission Apte et al. (2013).

While the role of PSCs in advanced pancreatic cancer is now well-accepted, evidence is also accumulating to suggest that PSCs may be activated at the earliest stages of pancreatic carcinogenesis, i.e., around PanIN lesions. Pandol et al. (2012) have described a distinct stromal reaction comprising extensive collagen deposition and α-SMA positive activated PSCs around PanIN lesions (Figure 5) which eventually lead to overt pancreatic cancer in a mouse model overexpressing Kras^G12D^. Similarly periostin (solely expressed by PSCs) has been observed in intraductal papillary mucinous neoplasms of the human pancreas (Fukushima et al., 2008), further supporting the idea that PSCs are activated early in the neoplastic process. Recent *in vitro* studies have confirmed an interaction between PanIN cells and PSCs. Exposure of PSCs to PanIN cells isolated from Kras^G12D^ mice significantly increased PSC proliferation, activation (α-SMA), fibronectin synthesis, and MMP expression (Pandol et al., 2012), indicating that preneoplastic cells have the capacity to activate PSCs in the early stages of carcinogenesis.

Based on findings reported by Funahashi et al. (2007), reciprocal effects of PSCs on PanIN cells which could facilitate progression to overt PDAC may also be postulated. The authors have shown that nimesulide, a selective inhibitor of COX-2 (which as noted earlier, is expressed by PSCs and implicated in PSC-cancer interactions), retards the progression of pancreatic cancer precursor lesions in a GEM model.

## Therapeutic targeting of stroma in pancreatic cancer

Clinical outcome in pancreatic cancer has not improved significantly over many decades. The usual regimens of surgery, radiotherapy, and chemotherapy benefit only a small minority of patients, and even in these patients, the chances of recurrence and emergence of chemoresistant cancers are high. The majority of patients are either not suitable for surgery at diagnosis or develop resistance to single chemotherapeutic agents. In a bid to address drug resistance, combination therapies have been trialed where the standard chemotherapeutic agent gemcitabine is combined with other agents such as Folfirinox (comprising 5-fluorouracil, leucovorin, irinotecan, and oxaliplatin) or with targeted drugs such as growth factor inhibitors or with soluble taxanes such as Abraxane. The most recent studies combining gemcitabine with Abraxane (nab-Paclitaxel) (Von Hoff et al., 2013) or Folfirinox (Conroy et al., 2011) have reported an increase in overall survival, but the benefit is marginal (a few months increased survival). Thus, it is clear that a new approach is required to improve the prognosis of this disease.

For reasons already discussed, strategies are now being developed to target not only cancer cells but also the desmoplastic reaction, and initial studies have been focused on ways to inhibit PSC activation.

One of the signaling factors known to mediate PSC activation is the Hedgehog pathway (which is essential for embryonic development, but usually not detectable in adult healthy pancreas) (Bailey et al., 2008). This pathway has been also been implicated in stem cell regulation and neoplasia (Thayer et al., 2003). Binding of the Hedgehog ligand (Sonic, Indian, and Desert Hedgehog) to its receptor Patched releases the co-receptor Smoothened from repression and results in translocation of the transcription factor Gli-1 from the cytoplasm to the nucleus where it regulates genes involved in cell differentiation, proliferation, apoptosis, adhesion, and migration. Abnormal activation of Hedgehog pathway has been reported in several cancers including basal cell carcinoma, lung, prostate, and pancreatic cancer. Inhibition of Smoothened by cyclopamine or its derivative IPI-926 in transgenic models of pancreatic cancer has been recently studied (Feldmann et al., 2007; Olive et al., 2009). Cyclopamine marginally increased median survival by 6 days, while treatment of mice with IPI-926 in combination with gemcitabine, resulted in increased delivery of the chemotherapeutic agent to cancer cells, but had only a transient effect on improved blood vessel density and extension of median survival. Subsequently, Hwang et al. (2012) used another Smoothened inhibitor AZD8542 in an orthotopic model of pancreatic cancer produced by implantation of a mixture of PSCs and cancer cells in the pancreas. AZD8542 was reported to reduce tumor volume, metastasis, and Hedgehog downstream signaling activity. Based on these encouraging pre-clinical reports, clinical trials using Hedgehog inhibitors were commenced. Unfortunately the phase II trial with IPI-926 had to be abandoned prematurely due to decreased survival of patients in the treatment arm. The lack of translation of the preclinical findings to the clinical setting may reflect the fact that preclinical models do not fully capture the heterogeneity of human pancreatic cancer, or that the preclinical findings need to be better confirmed using a range of experimental settings.

Taxanes such as Paclitaxel and Docetaxel have been used as chemotherapeutic agents in a variety of cancers. The compounds act by preventing microtubule depolymerization and interfering with the cell cycle, but their use is hampered by their toxicity and insolubility in water. Nanoparticle albumin complexed paclitaxel (nab-paclitaxel) was developed to overcome the issues of solubility and to enhance drug delivery through albumin facilitated receptor-mediated transcytosis (Yardley, 2013). Administration of nab-paclitaxel alone or in combination with gemcitabine in a patient-tumor-derived subcutaneous xenograft model depleted the stroma in the tumors and increased perfusion via an increase in blood vessel diameter with consequent improved delivery of gemcitabine to tumor cells (Von Hoff et al., 2011). The mechanisms mediating the effects of nab-paclitaxel on the stroma are unknown. However, with regard to the anti-cancer effects, it is postulated that the albumin in nab-paclitaxel is bound by secreted protein acidic and rich in cysteine (SPARC), an albumin binding glycoprotein that is overexpressed in pancreatic cancer stroma (Neuzillet et al., 2013), leading to accumulation of nab-paclitaxel near tumor cells (Yardley, 2013). Furthermore, nab-paclitaxel may increase the availability of gemcitabine within tumor tissue by inducing the generation of reactive oxygen species within cancer cells, leading to inhibition of cytidine deaminase and consequently decreased metabolic inactivation of gemcitabine (Yardley, 2013). As noted earlier, a recent Phase 3 trial has compared the effects of nab-paclitaxel plus gemcitabine to gemcitabine alone in patients with metastatic pancreatic cancer (Von Hoff et al., 2013). The combination was found to significantly improve overall survival as well as progression-free survival compared to gemcitabine alone (8.5 vs. 6.7 months and 5.5 vs. 3.7 months, respectively). Although the improvements may be regarded as modest, the results support the concept that targeting the stroma in addition to cancer cells may be a potentially beneficial approach.

With regard to targeting the immune cells in PDAC stroma, Beatty et al. (2011) have demonstrated in the KPC mouse model of pancreatic cancer that activation of CD40, a member of the TNF receptor superfamily, activates macrophages in the stroma and results in apoptosis of cancer cells as well as a reduction in stromal collagen. Activation of CD40 was achieved by systemic administration of a CD40 agonist monoclonal antibody to KPC mice. Using a similar approach in a Phase I study in a small number of chemotherapy-naïve advanced pancreatic cancer patients, the authors have reported that the antibody in combination with gemcitabine was well-tolerated with some evidence of anti-tumor activity, but with heterogeneous responses particularly with regard to metastatic lesions (Beatty et al., 2013). Thus, larger randomized controlled trials will be needed before the role of a CD40 agonist monoclonal antibody in pancreatic cancer treatment can be clearly determined.

Other compounds that have been used to target the stroma, but so far only in preclinical models, include:

- Angiotensin II receptor antagonists: Angiotensin II, a component of the renin-angiotensin system, has been shown to induce PSC proliferation, ECM synthesis and migration, and to increase the production of growth factors by PSCs. Thus, angiotensin II receptor blockade, already in clinical use in hypertension, has been recently assessed in a subcutaneous xenograft model of pancreatic cancer. Using the inhibitor olmasartan, Masamune et al. (2013) report a significant decrease in primary tumor growth accompanied by decreased α-SMA staining and ECM production in mice injected with a mixture of PSCs and cancer cells, but not in mice injected with cancer cells alone. Similarly, using losartan (another Angiotensin II receptor inhibitor), Chauhan et al. (2013) have reported decreased αSMA positive cells, and reduced collagen and hyaluronan production in the stroma of pancreatic cancer in an orthotopic mouse model.
- Pirfenidone (a pyridone compound known to be an effective antifibrotic agent in idiopathic pulmonary fibrosis): Treatment with this compound using subcutaneous and orthotopic models of pancreatic cancer has been reported to decrease the growth of tumors produced by the injection of a mixture of pancreatic cancer cells and PSCs, but not that of tumors produced by cancer cells alone (Kozono et al., 2013). *In vitro* studies showed that pirfenidone inhibited PSC proliferation, invasion, and migration, and interrupted the interaction between pancreatic cancer cells and PSCs; these effects were associated with decreased expression of PDGF-A, HGF, periostin, collagen type I, and fibronectin in PSCs, as well as reduced PSC activation (decreased α-SMA expression) (Kozono et al., 2013). The findings suggest that pirfenidone regulates PSC function and inhibits cancer growth.
- PEGylated human recombinant PH20 hyaluronidase (PEGPH20): This compound enzymatically degrades one of the predominant components of the ECM, hyaluronan. PEGPH20 treatment of KPC mice resulted in stromal depletion and decompression of tumor vessels leading to an increase in tumor vascular patency without increasing vessel density. PEGPH20 also increased fenestrations in endothelia and interendothelial junction gaps that increased the permeability of the endothelium to macromolecules. When combined with gemcitabine, PEGPH20 treatment improved the delivery of gemcitabine to tumor cells inhibiting tumor growth and extending the median survival of the mice (Provenzano et al., 2012; Jacobetz et al., 2013).
- Phytonutrients ellagic acid and embelin: Ellagic acid is a polyphenol found in a variety of nuts and fruit, while embelin is a phytochemical from a Japanese herb Arsidae Japonicae. These compounds have been reported to decrease proliferation and increase apoptosis of cancer cell as well as stellate cells resulting in significantly reduced tumor volumes in a xenograft model of pancreatic cancer (Edderkaoui et al., 2013).

In conclusion, it is now abundantly clear that the prominent stromal/desmoplastic reaction of pancreatic cancer can no longer be dismissed as a mere epiphenomenon of carcinogenesis. Indeed, available evidence strongly indicates that this stromal reaction, and in particular the cells responsible for its production, PSCs, likely play a key role at the earliest stages of pancreatic cancer development. Therefore, all components of this reaction (stromal cells and collagenous matrix) warrant attention as potentially useful, additional therapeutic targets in this disease. The challenge in this field of research will be to ensure that preclinical testing is carried out with experimental models (or a range of models) that not only closely simulate the pathology, but also account for the heterogeneity of human pancreatic cancer, so as to successfully translate research findings into clinically effective therapies.

### Conflict of interest statement

The authors declare that the research was conducted in the absence of any commercial or financial relationships that could be construed as a potential conflict of interest.
